# A case report of the rare fifteen-and-a-half syndrome

**DOI:** 10.1097/MD.0000000000014553

**Published:** 2019-03-22

**Authors:** Mengmeng Li, Xuan Li, Lina Liu, Mingsu Zhu, Dandan Lu, Pei Wang

**Affiliations:** a Department of Neurology, Baoding No. 1 Central Hospital; b Anxin County Hospital, Baoding, Hebei, China.

**Keywords:** bilateral peripheral facial paresis, fifteen-and-a-half syndrome, infarction, one-and-a-half syndrome, pontine tegmentum

## Abstract

**Rationale:**

The dorsal tegmentum of the caudal pons, including the medial longitudinal fasciculus (MLF), the paramedian pontine reticular formation (PPRF), abducens nucleus, and the adjacent facial nerve is the anatomical basis of the the fifteen and a half syndrome (15½) syndrome. No patients of 15½ Syndrome presenting with bilateral peripheral facial paralysis and one-and-a-half simultaneously at the onset have been reported up to now.

**Patient concerns:**

A 54-year-old woman complained of diplopia, slurred speech, and slightly distal numbness of the left upper limb for 4 days in our hospital.

**Diagnoses:**

The diffusion weighted image (DWI) and apparent dispersion coefficient (ADC) of MRI revealed the causative lesion in pons including bilateral pontine tegmentum and a narrow lesion along the midline in the right of the pons. Her clinical manifestations with results of MRI resulted in the diagnosis of the fifteen-and-a-half-syndrome.

**Interventions:**

The patient received antiplatelet aggregation, plaque stabilization, free radicals elimination, circulation improvement, nerves nourishment, and other symptomatic treatments.

**Outcomes:**

Two months later, her ocular movement recovered, and the bilateral facial paresis showed some improvement.

**Lessons:**

First, our patient with 15½ syndrome maybe one of mutants whose bilateral pontine tegmentum is supplied by unilateral pontine paramedian perforator artery. Second, DWI combined with ADC may be applied in the diagnosis of fifteen-and-half syndrome when the lesions of infarction are too small to be revealed by MRI scan.

## 1 Introduction

**Figure 1:**
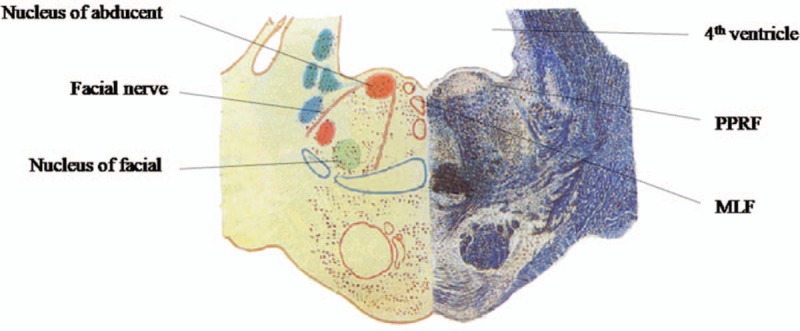
Transverse section of the pons through the facial coliculus.

Fifteen-and-a-half syndrome (15½ syndrome), including one-and-a-half syndrome and bilateral peripheral facial paresis, was firstly named by Bae and Song^[1]^ in 2005. The dorsal tegmentum of the caudal pons including the medial longitudinal fasciculus (MLF), the paramedian pontine reticular formation (PPRF), abducens nucleus, and the adjacent facial nerve is the anatomical basis of the rare syndrome (Fig. 1). It is necessary to improve the clinical recognization of this rare syndrome. So far, there are few reports about 15½ syndrome. Here, we report a patient who complained of diplopia, slurred speech and slightly distal numbness of the left upper limb was diagnosed the 15½ syndrome finally. After treatment, the patient gradually recovered. Two months later, the patient was followed-up and the prognosis was relatively good.

## 2 Case report

**Figure 2:**
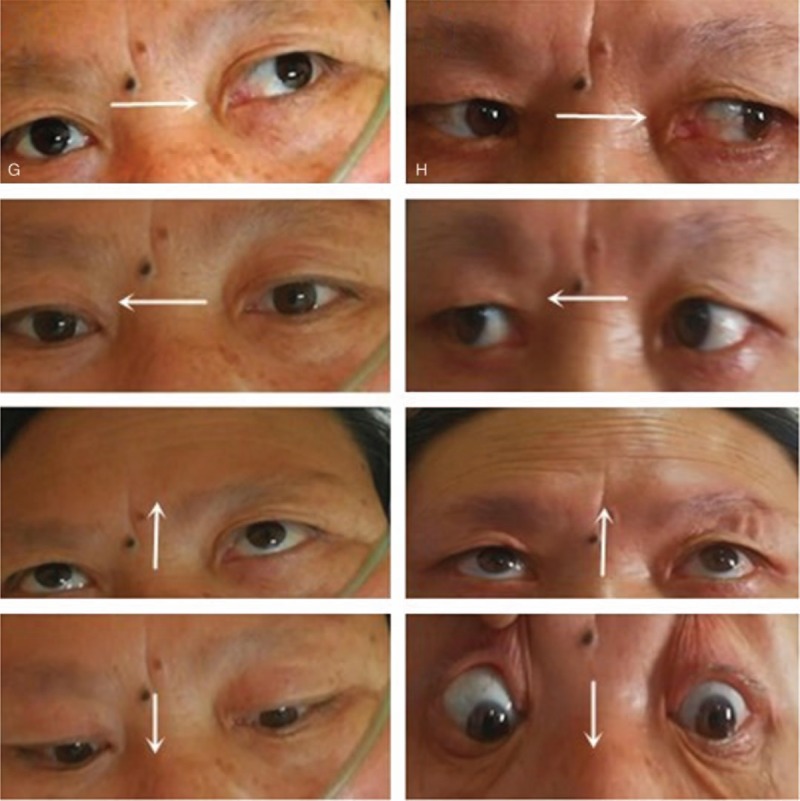
According to the left half of the picture (A), the right conjugate gaze palsy, loss of adduction of the right eye and limited abduction of left eye were observed on admission. Staring to the left, there was horizontal nystagmus. The rest half picture (B) demonstrated the obvious frontal striae and eye movement disorder recovered substantially in 3 months after admission.

**Figure 3:**
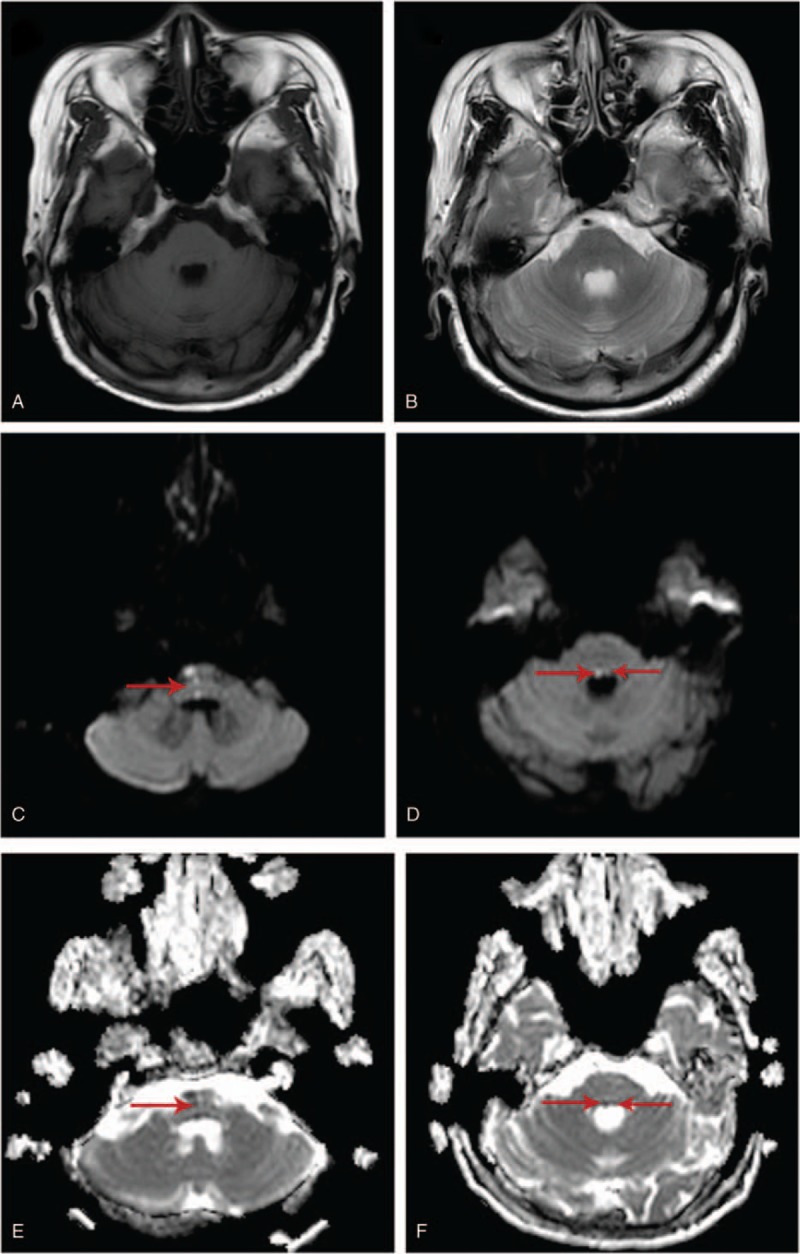
Axial T1-weighted (A) and T2-weighted (B) images was consistent with no definite abnormality. Axial diffusion-weighted image (C and D) demonstrated the restricted diffusion in the territory of bi-lateral pontine tegmentum and another bigger lesion in the right of the pons near the midline, whose apparent diffusion coefficient (ADC) was low, meanwhile. ADC = apparent diffusion coefficient.

A 54-year-old right-handed woman was admitted to our hospital presenting with diplopia, slurred speech, and slightly distal numbness of the left upper limb for four days. Her medical history was notable for hypertension, type 2 diabetes mellitus, hysteromyomectomy and ischemic stroke without significant consequences. In the neurological examination, the following symptoms were found: dysarthria, bilateral seventh nerve palsy, right conjugate gaze palsy, loss of adduction of the right eye. Besides, horizontal nystagmus on abduction of the left eye was also observed. In addition, the patient showed slightly limited abduction of left eye (Fig. 2A). Brain MRI showed the infarctions including the bilateral pontine tegmentum and a narrow lesion along the midline in the right of the pons, which run through the pontine tegmentum to the ventral side (Fig. 3). After admission, the patient received treatments such as antiplatelet aggregation, plaque stabilization, free radicals elimination, circulation improvement, nerves nourishment, and other symptomatic treatments.

In the period of 2 months of follow-up, the patient's slurred speech improved definitely. And her ocular movement recovered basically. Besides the bilateral facial paresis was also improved (Fig. 2H).

## 3 Discussion

One-and-a-half syndrome referred firstly by Fisher in 1967, may result from lesions in the abducens nucleus, in the abducent nerve and MLF, or PPRF.^[2]^ Before long, Eggenberger^[3]^ discovered the combination of one and a half syndrome and ipsilateral facial nerve palsy, and called it as eight-and-a half-syndrome.

In 2005, Bae and Song^[1]^ and Song examined a 67-year-old man with one-and-a-half syndrome and a bilateral lower motor neuron facial, and named a novel syndrome—The 15½ Syndrome (7 + 7 + 1½). They attributed these symptoms to a bilateral tegmental pontine lesion involving the right abducens nucleus or fasciculus, bilateral or right medial longitudinal fasciculus, and bilateral intraaxial fasciculei of the facial nerve. In our patient, her right conjugate gaze palsy and internuclear ophthalmoplegia on left gaze, and bilateral facial paresis accorded with the 15½ Syndrome.

The lesion of pons tegmentum can be found in the patients of stroke, multiple sclerosis, tumor, and vasculitis.^[4]^ Eight-and-a-half syndrome is the typical representative.^[5–6]^ However, only a few reported 15½ Syndrome cases hitherto have been reported in patients with cerebral infarction only. And in these cases, the time of peripheral facial paralysis occurs at different times. The aggravation of the disease may be related to the enlargement of infarction and edema. However, no cases of simultaneous peripheral facial paralysis in the 15½ Syndrome have been reported up to now. Our case is so nontypical in both course of disease and image revelation of MRI that it may be pioneering in the above-mentioned fields.

The dorsal tegmentum of the caudal pons including the medial longitudinal fasciculus (MLF), the paramedian pontine reticular formation (PPRF), abducens nucleus and the adjacent facial nerve is the anatomical proof of the 15½ syndrome (Fig. 1). The causative lesion of the woman patient which was revealed by DWI and ADC (Fig. 3) demonstrated the ischemic infarction in the right paramedian infarctions of the pontine near the midline and bilateral pontine tegmentum, while the T1-weighted and T2-weighted images were still negative. It can therefore be concluded that the T1-weighted and T2-weighted images of MRI scan are difficult to find the lesions when them are small. And DWI combined with ADC can help the diagnosis. In addition, some scholars put forward the point that the small lesions in the stem may be “no abnormality” in the early onset of the ischemic stroke, and the timely DWI reexamination or even thin layer scan may assist the diagnosis.

Normally, the blood supply of the tegmentum on one side of the pontine originates from the deep perforating branch of basilar artery and pontine tegmentum branch of the superior cerebellar artery, which usually branches symmetrically and supplies the unilateral pontine tegmentum, but there is also vascular anatomical variation that the bilateral pontine tegmentum is supplied by unilateral pontine paramedian perforator artery. Fisher^[7]^ discovered that a paramedian artery on one side, on reaching the region of the MLF on its side, is divided into 2 almost equal-sized branches, one of which crosses the midline to run to the region of the MLF on the contralateral side, and the other branch stays on its own side and runs to the ipsilateral MLF. The ratio of such dividing to nondividing paramedian tegmental pontine branches is estimated at 1:7. We therefore speculate that the patient may be one of the rare mutants. Given the unclear cause of our case and the 15½ syndrome is very rare, we hope this case report will be helpful in the diagnosis. In addition, longer term follow-ups needed to evaluate the effectiveness of the treatment and monitor the recurrence.

## Acknowledgment

On behalf of all the authors, I acknowledge the contribution of Professor Zhaihui Wang to this article.

## Author contributions

**Formal analysis:** Mengmeng Li, Xuan Li, Lina Liu.

**Investigation:** Mingsu Zhu.

**Supervision:** Dandan Lu, Pei Wang.

**Writing – original draft:** Mengmeng Li.

Pei Wang orcid: 0000-0002-0079-6163.
